# The role of the poultry industry on kidney and genitourinary health in Pakistan

**DOI:** 10.12669/pjms.36.ICON-Suppl.1718

**Published:** 2020-01

**Authors:** Salman Imtiaz, Ashar Alam, Beena Salman

**Affiliations:** 1 Prof. Dr. Salman Imtiaz Senior Consultant Nephrologist, Department of Nephrology, The Indus Hospital Karachi, Korangi Crossing 75190, Pakistan; 2 Dr. Ashar Alam Senior Consultant Nephrologist and Medical Director, Department of Nephrology, The Indus Hospital Karachi, Korangi Crossing 75190, Pakistan; 3 Dr. Beena Salman, Head of the Department of Biostatistics and Epidemiology, Department of biostatistics and epidemiology, Dorab Patel Post Graduate Training & Research Center, The Kidney Center Post Graduate Training Institute, Karachi, Pakistan

**Keywords:** Kidney failure, chronic kidney disease of unknown etiology, Chicken industry, Environmental diseases, Non-communicable diseases, Genitourinary diseases

## Abstract

Pakistan is experiencing a “double burden’’ of disease. Under-development is associated with illnesses like infections and nutritional deficiency, and is accompanied with diseases linked with development, such as diabetes, hypertension, cancer and chronic kidney disease. In Pakistan, renal and genitourinary diseases are an important, unaddressed health issues. Chronic kidney disease of unknown etiology (CKDu) is a recognized form of renal failure in Pakistan. A possible cause of CKDu is toxins such as arsenic, cadmium, lead and other heavy metals associated with renal and genitourinary diseases. The poultry industry is an important source of both heavy metal toxins and also mycotoxins spread in the process of farming. Of the numerous mycotoxins, zearalenone and ochratoxin are well-known for their hazardous effects on genitourinary and renal parenchyma respectively. We reviewed the literature using PubMed and Google Scholar databases for levels of these toxins in various constituents of chicken farming like chicken feed, meat, litter and human drinking water contamination in various parts of the country. We found that these toxins are in higher levels than recommended.

## INTRODUCTION

The global upsurge in non-communicable diseases (NCDs), including heart disease, diabetes, cancers and chronic lung diseases is such that the World Health Organization (WHO) has declared a “public health emergency.” NCDs cause 60% of global mortality annually and low- and middle-income countries (LMICs) account for 80% of this loss of life.^1^

The environment plays an important role in this rising trend. Early exposure to environmental risk factors, such as chemicals and air pollutants, might increase NCD risk throughout life.^2^ Growing evidence shows environmental factors affect the increasing prevalence of chronic kidney disease (CKD),^3^ especially in LMICs. Direct effects on the kidney may occur via water and food contamination by industrial toxins, and indirectly through environmental invasion causing other NCDs which eventually cause renal failure.^4^

A recent worldwide study in 12 LMICs has reported CKD prevalence of 14.3% in the general population and 36.1% in high-risk groups.^5^ In Pakistan, Ashar and Farhana found overall CKD prevalence of 16.6% in the community in the cosmopolitan city of Karachi.^6^ CKD in Pakistan, as elsewhere, is mostly caused by diabetes mellitus and hypertension, followed by kidney stones, glomerulonephritis and chronic kidney disease of unknown etiology (CKDu).^7^ The increasing prevalence of CKDu in Pakistan is concerning. Multiple studies to identify a particular offending agent; to assess the affected geographical areas or explore NCD hotspots have been done.^8^ The incidence and prevalence of CKDu in Pakistani hospitals is 9.1% and 26.1% respectively.^9^,^10^ No community-based studies have been done so far.

Industrial toxins, particularly heavy metals like arsenic, cadmium, copper sulphate, lead, and various mycotoxins like zearalenone and ochratoxin are associated with renal and genitourinary diseases.^11^ The chicken and poultry farming may contribute to the spread of these heavy metals and mycotoxins. The poultry industry, largely unregulated and unsupervised, may therefore have a role in CKDu in Pakistan.^12^ In this literature review, we explored toxins in constituents of chicken farming in different parts of the country and draw links with renal dysfunction and disease.

### Search strategy

A keyword search of PubMed and Google Scholar was performed using “chronic kidney disease of unknown etiology”, “heavy metals and kidney disease”, “mycotoxin and kidney disease”, “poultry and kidney and genitourinary disease”. A manual search for relevant references from relevant articles not identified by the electronic search was performed. Only studies published in English until December 2018 were included.

### Poultry Industry in Pakistan

In the last decade, Pakistan has boosted the poultry industry with incentives and favorable policy decisions. Large scale investment has led to over 28,000 commercial poultry farms and 150+ feed mills in the country. About 2,821 million tons of chicken feed are produced annually and the public consumption has increased to 834 metric ton of chicken meat per annum.^13^,^14^ However, this expanding industry, unregulated by directives and surveillance, may be rapidly producing substantial health hazards.

**Fig.1:**
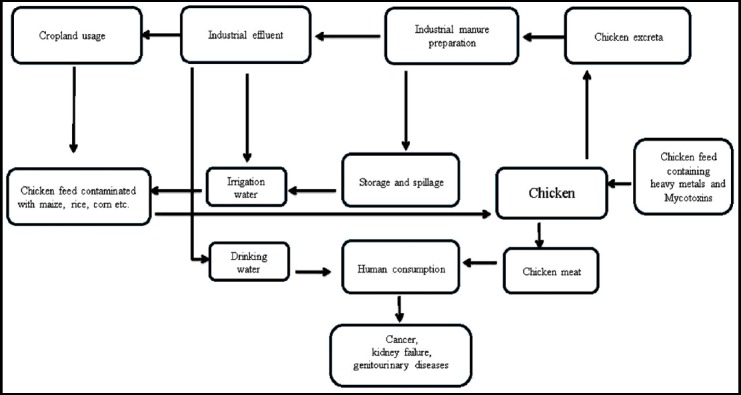
Schematic diagram showing pollution and contamination caused by chicken feed derived from Garbarino JR, Bednar A.^17^

The effect of these health hazards includes dangerous chemical and microbiological agents. An unacceptably high heavy metal content in ‘normal’ chicken feed or hazardous environmental exposure to mycotoxins during feed preparation has been shown (Table-I). Chickens are vectors in spreading these toxins through meat, feed, eggs and litter or manure, either directly or indirectly by polluting water and agriculture (Fig.1). This review will focus on three such contaminants i.e. arsenic, ochratoxin and zeralenone, which are important in causing kidney and genitourinary diseases.

**Table 1:** Potential chemical contaminants in poultry meat products.

Contaminant	Source	Potential adverse effects
Arsenic	Environmental contamination. Use of Arsenic-based anticoccidial agents.	Human carcinogen - inducing primary skin cancers. Nephrotoxic agent.
Cadmium	Environmental contamination	Nephrotoxic agent
Fluoride	Contamination of mechanically separated poultry with finely powdered bone.	Dental fluorosis
Lead	Environmental contaminant. Contamination of wild crafted birds such as the Magpie Goose.	Human neurodevelopmental toxin with children being particularly sensitive
Mercury	Contamination of poultry fishmeal starter rations. Contamination of wild crafted birds such as the Mutton bird.	Human neurotoxin - developing foetus particularly sensitive
Selenium	Contamination of poultry fishmeal starter rations.	Adverse effects on nervous system.
Polychlorinated biphenyls	Environmental contaminant.	Potential human carcinogen. Very low tolerable monthly intake.
Aflatoxin B1, B2, G1, G2	Aspergillus flavus, and A. parasiticus contamination of corn, peanuts and other feed ingredients	Aflatoxin B1 – potential human carcinogen
Trichothecenes T-2 and HT-2 toxin Deoxynivalenol (DON) Vomitoxin	Fusarium graminearum, F. crookwellense and F. culmorum contamination of wheat, barley and corn	Acute food poisoning
Zearalenone	Fusarium graminearum, F. crookwellense and F. culmorum contamination of wheat and corn	Possible carcinogen – effects the reproductive system of laboratory animals and pigs
Ochratoxin A	Aspergillus ochraceus and Penicillium verrucosum contamination of barley, wheat and many other commodities	Nephrotoxin, possible human carcinogen
Fumonisin B1	Fusarium moniliforme plus several less common species contamination of corn	Nephrotoxin, possible human carcinogen

(Modified from Food Standards Australia New Zealand November 2005).

**Table II:** Levels of Arsenic, Ochratoxin and Zearlenone in different cities of Pakistan.

Cities and province	Arsenic(ppm)	Levels WHO Recommendation <10 ppm
Gumbet, Khairpur^53^	Arsenic(ppm)	45
Matiari^53^	Arsenic(ppm)	30-120
Tando Mohammad Khan^53^	Arsenic(ppm)	30-100
Hala^53^	Arsenic(ppm)	40-180
Nasarpur^53^	Arsenic(ppm)	13-80
Nosheroferoz^53^	Arsenic(ppm)	25-60
Sakrand^53^	Arsenic(ppm)	25-50
Sukkur^53^	Arsenic(ppm)	80
Dadu^53^	Arsenic(ppm)	5-60
Manchar Lake^49^	Arsenic(ppm)	35-157
Karachi^52^	Arsenic(ppm)	80
Feroz wala^48^	Arsenic(ppm)	45
MuridKe^48^	Arsenic(ppm)	40
Shekhopura^48^	Arsenic(ppm)	65
Safdarabad^48^	Arsenic(ppm)	55
Multan^54^	Arsenic(ppm)	60-1000
Kalan wala^54^	Arsenic(ppm)	32-1900
MuzafarGarh^54^	Arsenic(ppm)	0-400
Punjab province^36^	Ochra toxins(mc/kg)	5-84.5
Punjab province^39^	Zeralenone(ppm)	19.45

### ARSENIC:

***a) Mode of spread:*** Arsenic occurs naturally in both organic and inorganic forms. Organic arsenic compounds have been used in the poultry industry for years. Roxarson (Ristat; 3-nitro, 4-hydroxy-3-nitrobenzenearsonic acid) is an arsenic-containing component of chicken feed which promotes growth, improves efficiency of feed utilization and increases chicken meat pigmentation. It also controls spread of coccidiosis, a protozoal disease that is a major killer of farmed chickens.^15^ However, over 90% of arsenic is excreted unchanged in the feces. Improper decontamination of this leads to arsenic-rich manure for soil fertility. This subsequently contaminates cereals like maize, wheat and rice.^16^ Soil bacteria, especially *Clostridium* species, convert organic arsenic into dimethyl arsenic acid, monomethyl arsenic acid and inorganic arsenic compounds. These metabolites are more toxic than the original precursors found in the environment.^17^,^18^ Improper storage of chicken litter in uncovered areas also causes environmental exposure. During rainfall, arsenic leaches from storage and seeps into nearby rivers, stream, and wells.^19^

**Fig.2:**
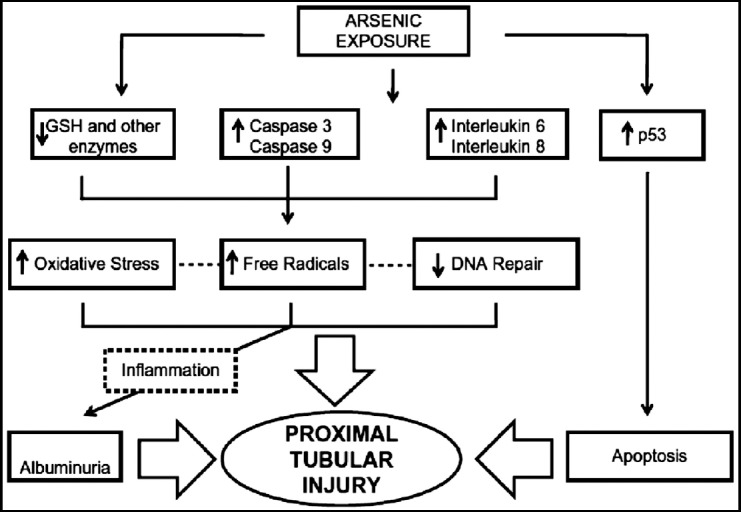
Mechanism of renal injury by Arsenic.^19^

***b) Mechanism of arsenic toxicity:*** The mechanism of arsenic-induced renal toxicity is well reported by Osorio and Silva in a state of the art review.^20^ Arsenic affects the proximal convoluted tubules by depleting intracellular glutathione stores. This activates the caspase-3 and -9 signaling pathway, thereby increasing expression of interleukin-6 and interleukin-8 and causes activation of the p-53 apoptotic pathway. There is increased production of Reactive Oxygen Species (ROS) and other free radicals, along with inflammation and apoptosis. Additionally, arsenic uncouples oxidative phosphorylation causing reductions in sodium, phosphate and glucose transport manifesting clinically as Fanconi syndrome causing phosphaturia, glucosuria and low-molecular weight proteinuria (Fig.2).

***c) Effect of arsenic on chickens*** Kaazi and Shah evaluated arsenic translocation from chicken feeds to different tissues of broiler chicken and their excretory products. They collected samples of chicken feed, broiler chicken meat and manure from poultry farms in Hyderabad, Pakistan. In all three types of samples, levels of arsenic exceeded international recommendations.^21^

To investigate if chicken feed is a source of chicken meat contamination has recently been evaluated at the Pakistan Council of Scientific and Industrial Research Laboratory. The basic constituents and additives in chicken meat, feed and droppings in broiler and organic chickens were compared. Broiler chicken were fed on commercial feeds while organic chickens were given natural grains and grit and roamed free. High levels of Roxarson were found in broiler chicken meat and their droppings, not seen in the organic chicken group.^22^ In another study, Ghani and Sahito examined the levels of arsenic and lead in food items, including chicken meat. They found high arsenic concentrations, ranging above 41.3 ng/g, over twice the tolerated weekly intake of 15µg/g body weight.^23^

The impact of this contamination has been analyzed at both cellular and histological level. At the University of Agriculture, Faisalabad, Mashkoor and Khan compared the pathological effects of arsenic (50 mg/kg) on the blood and body tissues of 90-day broiler chickens. They found a significant reduction in erythrocyte count, hemoglobin and packed cell volume in arsenic treated chickens compared with controls. The liver showed gross parenchymal degeneration and necrotic changes. There were intranuclear changes like hyperchromasia, pyknosis, fragmentation and cells devoid of nucleus. The kidneys were worse affected with moderate to severe necrosis of tubular epithelial cells characterized by pyknotic nuclei and cytoplasmic vacuolation. Glomeruli were atrophied with massive lobulation and fragmentation of capillary membrane along with mononuclear infiltration and increased urinary spaces were observed.^24^

Although not yet evaluated in humans, malignant potential has been shown in animal studies where inorganic arsenic exposure has been associated with neoplastic transformation in both liver and kidneys. Continuous production of ROS and reactive nitrogen species (RNS) is thought to damage the thiamine nucleotides of DNA strands.^25^ Similarly, the mutagenic tendency of arsenic alone or in combination with copper sulphate (added to chicken feed for growth promotion, antifungal and anthelmintic effects) was compared at the University of Bahawalpur. Here, Ghaffar and Hussain observed the hematological and mutagenic effect in adult male birds fed different combinations and strengths of arsenic and copper sulphate versus a control, normal diet group. The frequency of erythrocytes with micronuclei, notched nuclei erythrocytes with nuclear remnant, blebbed nuclei, condensed nuclei and binucleated erythrocytes was significantly raised in the treated groups.^26^

**Fig.3:**
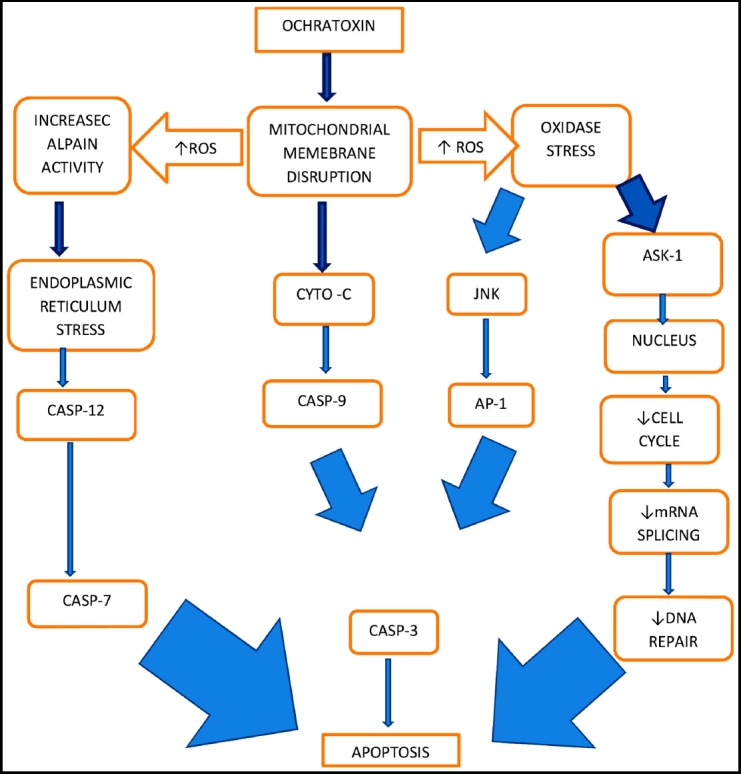
Mechanism of injury by Ochratoxin. Derived from mechanism of Ochratoxin Toxicity by Kőszegi T and Poor M.^43^

***b) Water Contamination with Arsenic:*** The Pakistan Council of Research in Water Resources evaluates arsenic levels regularly in the major cities of Pakistan.^27^ Persistently increasing arsenic contamination led to a national action plan initiated in 2007. A nationwide survey of drinking water was evaluated in 35 of 104 districts. Of 8712 samples, only 9% had arsenic levels meeting the WHO standard of 10 ppb. Almost 30% had levels above 10 ppb when validated by atomic absorption spectrometry and 77% had levels above 50 ppb. The highest values were seen in the Sindh province.^28^

Abbas and Cheema analyzed drinking water from Sheikhupura District in Punjab. High levels of arsenic were found in hand pump samples (76.22 ± 20.73 ppb) and were only just acceptably lower in bottled water (7.742 ± 3.066 ppb).^29^ In Sindh, Arain et al found high levels of arsenic (96µg/l) in ground water and 157µg/l in surface water.^30^,^31^ In Manchar Lake, the largest reservoir of fresh water in Pakistan, the levels were in the range of 35.2-158 µg/L, surpassing. the limit by 3-15 fold. In Jamshoro, Sindh the highest content of arsenic in surface water samples was found to be 50µg/l.^32^-^35^

The poultry industry may contribute to water contamination due to unregulated dumping of chicken feed and manure in drinking water resources. Further study is required to establish these possible links through community based studies.

***d) Effect of Arsenic on Human Kidney:*** There is evidence of the effects of arsenic on in the renal system of humans. A case report of an African-American woman with high urea and creatinine and proximal tubular abnormalities revealed a dietary history of approximately 20 ounces of chicken, five to six times a week. Her 24-hour urine collection showed arsenic concentrations of 126µgms whilst the threshold for toxic values is 50µ/24hrs.^36^

Hsueh et al found a dose-dependent relationship between total urinary arsenic levels and associated CKD, especially when levels were over 20.74 and creatinine levels less than 11.78 µg/g (OR 4.34; 95% confidence interval, 1.94-9.69). Those with high urinary total arsenic level or with low proportion of dimethylarsinic acid had a positive association with CKD when their plasma lycopene (an antioxidant which protects against ROS) level was low.^37^

Exposure to arsenic concentration in drinking water more than 300 microg/L is well known to cause renal and other systemic diseases, but the effect of mild to moderate exposure (10 to 100 micrg/L) is not known. In Michigan (USA), Meliker et al investigated the relationship between moderate arsenic levels and selected disease outcomes and found a significant association between the arsenic exposure and CKD.^38^

### Status of Mycotoxin in Pakistan

“Mycotoxins” refers to poisonous substances produced by fungi. Contamination of food commodities by mycotoxins is a significant food safety challenge^39^ causing a huge food wastage. Over 400 mycotoxins are recognized, from which aflatoxin, ochratoxin A (OTA), fumonisins, zearalenone (ZEA), deoxy nivalenol (DON) are important for their serious hazards on human and animal health. The mechanism of spreading and proliferation of different mycotoxins has been reviewed in detail elsewhere.^39^

### Zearalenone

Mycoestrogen or zearalenone (ZEA) is a metabolite of *Fusarium* species like Fusarium graminearum, a common contaminant of cereals and animal feed worldwide. It has structural similarity with 17 beta-estradiol and action on the hypothalamus and pituitary is similar to estrogen. It is well-known for deleterious effect on the human reproductive system^40^,^41^ e.g. early onset puberty in female children.^42^,^43^ The cellular effects of ZEA are poorly understood. Lysosomes may be the primary target as lysosomal injury may precede DNA strand breakage when explored in HEK293 cells (transformed human primary embryonic kidney cells).^44^

When 865 poultry feeds from different parts of Pakistan were investigated, 49% were contaminated with ZEA.^45^ Corn in chicken feed is thought to be the major source of ZEA contamination. When Khatoon et al evaluated ZEA concentration in maize throughout Pakistan, they found it was significantly higher than recommended levels.^46^ Iqbal and Rabbani analyzed 237 Pakistani breakfast cereals and found that 53% had ZEA contamination, 8% above the European Commission upper limit of 50 ug/kg.^47^ Iqbal and Zia found similar results when they examined 107 chicken feed and ingredients. Samples from farmers, industries and general stores from big cities in Punjab found poultry feed to have the highest levels of ZEA.^48^

In the only animal study conducted using chicken meat and feed in Pakistan, Sara Ahmed at Baqai University investigated the effects on estradiol, a zearalenone metabolite, in Wister rats was seen. One group was served control feed (rat chow), another served commercially prepared chicken feed and the third was given raw commercial boneless chicken meat. Results showed the gains in weight and estradiol level were highest among the rats fed chicken meat, followed by those fed chicken feed compared with controls.^49^

### Ochratoxin

Ochratoxins, another class of mycotoxins, are produced by Aspergillus and Penicillium species. Of three forms A, B and C, ochratoxin A (OTA) is most toxic. It is a powerful nephrotoxin, carcinogen, teratogen and immune-toxin^50^. Poultry is recognized as a major food source contaminated with ochratoxin.^51^

The mechanism by which ochratoxin causes cell damage is complex. In summary, it is thought to cause inhibition of protein synthesis and energy generation, possibly via oxidative stress, DNA adduct formation with apoptosis and necrosis causing cell cycle arrest. An excellent review has been written by Koszegi and Poor^52^ from which a schematic mechanism is extracted for the interested reader (Fig.2).

In Pakistan, Fareed and Khan evaluated levels of ochratoxin in poultry feed. Ochratoxin contamination was seen in 63% of poultry feed ingredients and 29% of poultry feed products.^53^ Abid and Khatoon analyzed OTA in mixed poultry feed and their ingredients from farms and markets in Lahore. Samples were collected in three time intervals, i.e. July to October (hot and humid), November to February (winter) and March to June (moderate). The incidence of OTA-positive samples was highest in July to October (82%), followed by March to June (67%) and lowest during November to February (58%). They concluded that OTA levels in poultry feed ingredients (e.g. canola, soybean and sunflower meal, maize and whole cotton seeds) were alarmingly high. Entry of mycotoxins into the poultry feed chains is irreversible. Strict regulation of feed and feed ingredients to prevent its spread in poultry is recommended.^54^

## CONCLUSION

Industrial pollution is an important cause of NCDs amongst which CKD has a dramatically increasing prevalence. CKDu, common in Pakistan, may result from exposure to toxin contaminated water and food and is a potentially preventable cause. Access to quality healthcare may be poor, resulting in late presentation of end stage kidney disease. The costs of care of CKD is extremely high, as is the mortality. There is an urgent need for evaluation, monitoring and regulation of the chicken and chicken feed industry to prevent mycotoxin, arsenic and heavy metal toxicity. Further community-based studies to link these toxins to CKDu are desperately required.
